# Distinct inflammatory signatures of upper and lower body adipose tissue and adipocytes in women with normal weight or obesity

**DOI:** 10.3389/fendo.2023.1205799

**Published:** 2023-06-26

**Authors:** Ioannis G. Lempesis, Nicole Hoebers, Yvonne Essers, Johan W. E. Jocken, Rosemary Dineen, Ellen E. Blaak, Konstantinos N. Manolopoulos, Gijs H. Goossens

**Affiliations:** ^1^ Institute of Metabolism and Systems Research (IMSR), College of Medical and Dental Sciences, University of Birmingham, Birmingham, United Kingdom; ^2^ Department of Human Biology, NUTRIM School of Nutrition and Translational Research in Metabolism, Maastricht University Medical Centre^+^, Maastricht, The Netherlands, Maastricht, Netherlands; ^3^ Centre for Endocrinology, Diabetes and Metabolism, Birmingham Health Partners, Birmingham, United Kingdom

**Keywords:** adipose tissue, adipokines, inflammation, body fat distribution, obesity

## Abstract

**Introduction:**

Upper and lower body fat accumulation poses an opposing obesity-related cardiometabolic disease risk. Depot-differences in subcutaneous adipose tissue (SAT) function may underlie these associations. We aimed to investigate the inflammatory signatures of abdominal (ABD) and femoral (FEM) SAT in postmenopausal women with normal weight or obesity.

**Methods:**

We included 23 postmenopausal women with normal weight (n = 13) or obesity (n = 10). *In vivo* secretion of adipokines from ABD and FEM SAT was measured using the arterio-venous balance technique. Adipokine gene expression and adipocyte morphology were examined in ABD and FEM SAT. Furthermore, adipokine expression and secretion were investigated *in vitro* using differentiated human primary ABD and FEM subcutaneous adipocytes derived from the study participants.

**Results:**

Plasma leptin and plasminogen activator inhibitor (PAI)-1 concentrations were higher, and ABD and FEM adipocytes were larger in women with obesity than normal weight. No differences in adipocyte size and blood flow were apparent between ABD and FEM SAT. We found significant release of leptin and monocyte chemoattractant protein (MCP)-1 from ABD and FEM SAT, with higher fractional release of MCP-1 from ABD than FEM SAT. Gene expression of leptin, PAI-1, and tumor necrosis factor-α was lower in ABD than FEM SAT and higher in women with obesity than normal weight. In ABD adipocytes, interleukin-6, PAI-1, and leptin gene expression were higher, while adiponectin and dipeptidyl-peptidase-4 gene expression were lower than in FEM adipocytes. Finally, ABD adipocytes secreted less MCP-1 compared to FEM adipocytes.

**Discussion:**

These findings demonstrate that upper and lower body SAT and adipocytes are characterized by distinct inflammatory signatures in postmenopausal women, which seem independent of adipocyte size.

## 1 Introduction

Obesity is characterized by excessive accumulation of white adipose tissue (AT), which contributes to the development of insulin resistance and related cardiometabolic diseases (1–4). Body fat distribution is an important determinant of cardiometabolic derangements (5). Abdominal (ABD) obesity (upper body fat accumulation) is associated with an increased risk for insulin resistance, type 2 diabetes mellitus, cardiovascular disease, and all-cause mortality, while gluteofemoral (lower body) fat storage is associated with a more beneficial cardiometabolic risk profile for a given body mass index (BMI) in both men and women (1, 6–10).

ABD obesity is characterized by subcutaneous AT (SAT) and visceral AT accumulation, which are both related to cardiometabolic risk factors, dependent on factors such as sex and ethnicity (11–13). However, next to differences in body fat distribution, AT dysfunction is tightly linked to obesity-related complications (1, 2, 14). AT dysfunction is characterized by adipocyte hypertrophy, impaired lipid metabolism, decreased adipose tissue blood flow (ATBF), mitochondrial dysfunction, altered oxygenation, a state of chronic low-grade inflammation, and impaired adipokine expression/secretion (1–3, 15–17). Together, these impairments contribute to lipid spillover in the circulation, ectopic fat deposition, and low-grade systemic inflammation, collectively aggravating cardiometabolic disease development (1, 4, 5, 18–20). The predominant sequestration of lipids in lower body AT depots in premenopausal women seems to confer protection against the development of cardiometabolic diseases (4, 8, 21–23). In addition to AT depot-differences in lipid metabolism, differences in the inflammatory signatures between upper and lower body AT may contribute to the disease risk associated with a certain body fat distribution pattern (8).

Studies that have compared the inflammatory phenotype of upper and lower body SAT are scarce. Although no major differences in gene expression of inflammatory markers were previously found between ABD and gluteal SAT (23, 24), recent findings suggest that *in vivo* IL-6 release from gluteofemoral SAT may be lower than from ABD SAT in healthy men with normal body weight (23, 25). The latter findings might indicate that lower body SAT is characterized by a more beneficial inflammatory phenotype. Importantly, it remains to be established whether differences in the SAT depot-specific expression and secretion of (anti-)inflammatory factors exist in women as well as between people with normal weight and obesity.

Therefore, the present cross-sectional study aimed to investigate whether the expression and secretion of several well-known (anti-)inflammatory adipokines differ between upper and lower body SAT in postmenopausal women with normal weight or obesity. We hypothesized that the expression and secretion of proinflammatory factors are higher in upper body as compared to lower body SAT and adipocytes. To test our hypothesis, we compared the *in vivo* release of several adipokines across ABD and femoral (FEM) SAT, and investigated SAT depot-specific adipocyte morphology and adipokine expression in well-phenotyped postmenopausal women with normal weight or obesity. Furthermore, the expression and secretion of adipokines was examined *in vitro* using differentiated human multipotent adipose-derived stem (hMADS) cells derived from ABD and FEM SAT from the same individuals.

## 2 Materials and methods

### 2.1 Study design

A total of 23 healthy postmenopausal women (aged 50–65 years) with normal weight (BMI 18–25 kg/m^2^) or obesity (BMI 30–40 kg/m^2^) were recruited. All subjects underwent a medical evaluation during the screening visit (see **Supplementary Materials Methods**—Study design for details). The *in vivo* measurements were conducted at the University of Birmingham/Queen Elizabeth Hospital Birmingham (Birmingham, UK). The University of Birmingham Ethics committee and the UK Health Research Authority National Health System Research Ethics Committee approved the present study (approval no. 18/NW/0392). The study was performed according to the Declaration of Helsinki, and all participants provided written informed consent before taking part in the study procedures. The *in vitro* experiments and sample analyses were performed at Maastricht University Medical Center^+^ (Maastricht, the Netherlands).

Exclusion criteria were smoking, cardiovascular disease, type 2 diabetes mellitus, liver or kidney malfunction, any chronic medical condition requiring the use of medication known to affect body weight, glucose and/or lipid metabolism, use of anti-inflammatory agents (e.g., non-steroidal anti-inflammatory drugs, steroids) within 14 days prior to study start, planned blood donation 2 months prior to or after study completion, and marked alcohol consumption (>14 alcoholic units/week). Premenopausal or perimenopausal women, defined as either regular periods or a period within the last 12 months from screening date, were also excluded. Finally, individuals were excluded from the study if blood vessels were unsuitable for cannulation (i.e., too-small veins or arterial plaques).

Participants were asked to arrive at the Clinical Research Facility after an overnight fast, having avoided strenuous exercise and alcohol for at least 24 h, on three occasions. Each of these study visits took place within 1 week of the previous visit, separated by at least 2 days. Briefly, during the first visit, participants were screened, and an oral glucose tolerance test (OGTT) was performed. During the second visit, arterio-venous concentration differences across ABD and FEM SAT were assessed and blood flow in these fat depots was determined. During the third visit, a dual-energy x-ray absorptiometry (DXA) scan was performed to determine body fat percentage and body composition, and ABD and FEM SAT biopsies were collected. These measurements are explained in more detail in the next section.

### 2.2 *In vivo* measurements

#### 2.2.1 Screening

Body weight, height, waist (measured midway between the lower margin of the last palpable rib and the top of the iliac crest) and hip circumferences (measured at the level of the greater trochanters) were determined. Blood pressure and heart rate were measured using a standard oscillometric blood pressure monitor with an upper arm cuff. Next, we screened blood vessels in ABD and FEM SAT using ultrasound to determine whether veins would be suitable for cannulation. Finally, an OGTT was performed to exclude individuals with type 2 diabetes mellitus.

#### 2.2.2 Body composition

A dual x-ray absorptiometry (DXA) scan was performed after an overnight fast to determine body composition and body fat percentage (Lunar iDXA, GE Healthcare) (26).

#### 2.2.3 Arterio-venous concentration differences

Arterio-venous concentration differences of adipokines across the ABD and FEM SAT depots were assessed, as described previously (27, 28). Briefly, selective venous catheterization of one the branches of the superficial epigastric veins (draining ABD SAT) was performed (28–30). Next, a superficial branch of the great saphenous vein (draining FEM SAT) was cannulated (31). Finally, an arterial catheter was inserted into the radial artery. Blood samples were taken simultaneously from the three sites (arterial, ABD, and FEM) at two different time-points 60 min after the cannulation procedures (allowing participants to relax), separated by 30 min, under fasting conditions.

For the ABD SAT depot, veins located above the inguinal ligament, as determined by using the anterior superior iliac spine and the projected pubic symphysis as reference points, were identified. The SAT areas lateral of the umbilicus and between the lower end of the rib cage and the inguinal ligament were scanned with ultrasound (Philips CX50 Ultrasound, Bothell, USA) on each side to identify suitable veins for cannulation and ATBF measurements in the ABD SAT depot. After application of local anesthetic (lidocaine hydrochloride 1%), a 20-gauge central venous catheter was inserted with the Seldinger technique. Veins in FEM SAT that were suitable for cannulation and ATBF measurements were identified by scanning the inner aspect of the thigh, approximately halfway between the groin and the knee. A catheter (Venflon^®^) was placed and secured in place. Finally, an arterial catheter was inserted into the radial artery of the non-dominant hand using local anesthetic (1% lidocaine) and ultrasound guidance.

After completion of sample collection and blood flow measurements, all catheters were removed, and the study participants were given a meal. Due to the technical difficulties to cannulate the small veins in these SAT depots and to collect blood samples, we successfully completed the measurements and sample collection for nine women with normal weight and six women with obesity. Due to the limited number of paired blood samples draining ABD and FEM SAT for the individuals with normal weight and obesity, we decided to pool the data for all study participants per SAT depot to achieve sufficient statistical power to detect SAT depot-differences in adipokine release.

#### 2.2.4 Adipose tissue blood flow

Fasting ATBF was measured in ABD and FEM SAT using a Doppler ultrasound technique, as previously described (32). Briefly, the SAT areas lateral of the umbilicus and between the lower end of the rib cage and the inguinal ligament were scanned on each side to identify suitable veins for ATBF measurements in the ABD SAT depot. In the FEM depot, the great saphenous vein and its branches drain mostly FEM SAT. Suitable FEM veins for ATBF measurements were identified by scanning the inner aspect of the thigh, approximately halfway between the groin and the knee.

### 2.3 Biochemical analyses

During screening, blood samples were drawn to determine electrolytes, liver enzymes, full blood count, thyroid hormones, glucose, insulin, and HbA1c. Blood samples were collected into heparinized tubes, centrifuged at 4°C at 1,000g, and plasma was snap-frozen and stored at −80°C until analysis. Adipokine concentrations were determined using high-sensitive ELISAs [adiponectin and PAI-1 from Biovendor, interleukin (IL)-6 and monocyte chemoattractant protein (MCP)-1 from Diaclone, and leptin and dipeptidyl-peptidase (DPP)-4 from R&D Systems, insulin MSD].

### 2.4 Adipose tissue biopsies and adipocyte morphology

ABD and FEM SAT biopsies and adipocyte morphology were collected and assessed, respectively, as described before (33). ABD SAT needle biopsy specimens (up to ∼1 g) were collected 6–8 cm lateral from the umbilicus and from the FEM region (anterior site of the upper leg), respectively, under local anesthesia (1% lidocaine) after an overnight fast. Biopsy specimens were immediately rinsed with sterile saline, and visible blood vessels were removed with sterile tweezers. A small part of the SAT sample was fixed overnight in 4% paraformaldehyde and embedded in paraffin for histology. Another part was used for isolation of hMADS cells, as described before (33). The remaining tissue was snap-frozen in liquid nitrogen and stored at −80°C for gene/protein expression analysis.

Histological sections (8 μm) were cut from paraffin-embedded tissue, mounted on microscope glass slides, and dried overnight in an incubator at 37°C. Sections were stained with hematoxylin and eosin. Digital images were captured with the use of a Leica DFC320 digital camera (Leica, Rijswijk, Netherlands) at ×20 magnification (Leica DM3000 microscope; Leica). Computerized morphometric analysis (Leica QWin V3, Cambridge, England) of individual adipocytes was performed by measuring at least 200 adipocytes per sample.

### 2.5 Calculations

Adipokine release across ABD and FEM adipose tissue was assessed using the arteriovenous difference technique. Fractional release [FR = ((venous - arterial concentration)/arterial concentration) * 100%] was calculated for each adipokine using the concentration from SAT depot-specific blood samples. A positive FR value reflects the release of adipokines from SAT. All calculations were performed as described previously (28–30).

Indexes of pancreatic *β*-cell function and insulin resistance were calculated using the updated computer model-based homeostatic model assessment (HOMA) method (34).

### 2.6 Human primary adipocyte experiments

hMADS cells, an established human white adipocyte model (35), were obtained from ABD and FEM subcutaneous SAT. Cells were seeded at a density of 2,000 cells/cm^2^ and kept in proliferation medium for 7 days and thereafter in differentiation medium for 14 days. All experiments were performed on day 14 of adipogenic differentiation. Paired ABD and FEM adipocyte samples derived from nine women with normal weight and nine women with obesity were used for these experiments.

### 2.7 Adipose tissue and adipocyte gene expression analysis

Total RNA was extracted from all frozen SAT specimens (∼150 mg) and hMADS cells using a TRIzol reagent (Invitrogen, Breda, Netherlands), and SYBR-Green–based real-time PCRs were performed using an iCycler (Bio-Rad, Veenendaal, Netherlands; primer sequences are shown in **Supplemental Table 1**). Results were normalized to the mean of 18S ribosomal RNA.

### 2.8 Adipocytokine secretion measurement

The medium of the hMADS cells was collected over 24 h to determine adipokine secretion using high-sensitive ELISA. If necessary, samples were diluted with a provided dilution buffer from the manufacturer prior to the assay, which was performed in duplicates, according to the manufacturer’s instructions.

### 2.9 Statistical analyses

To assess whether there was significant release of adipokines from ABD and/or FEM SAT, we compared the fractional release value for each adipokine against zero release (that is, no net release). AT depot-differences in the secretion of adipokines and gene expression within women with normal weight and obesity were analyzed using Student’s paired t-tests (Wilcoxon signed rank tests in case data were not normally distributed), while differences between individuals with normal weight and obesity were determined using unpaired t-tests (Mann–Whitney test in case data were not normally distributed). GraphPad Prism version 8 for Windows was used to perform statistics, and p < 0.05 was considered as statistically significant. Data are presented as mean ± SEM.

## 3 Results

### 3.1 Subject characteristics

Participants’ characteristics are shown in **Table 1**. By definition, the BMI was higher in women with obesity compared to normal weight (both p < 0.001). Furthermore, waist and hip circumferences were significantly higher in women with obesity, while the waist-to-hip ratio was not statistically different between groups (p = 0.443). The sizes of all AT depots examined (visceral, ABD, and leg fat) were higher in women with obesity (all p < 0.001). In addition, women with obesity tended to have higher fasting insulin concentrations (p = 0.053). In line, Homeostasis Model Assessment 2–Insulin Resistance was higher in women with obesity compared with normal weight (p = 0.050).

**Table 1:** Anthropometric characterization and metabolic profile of participants.

	Normal weight (n = 13)	Obesity (n = 10)	p Value
Age (years)	56.6 ± 1.5	56.6 ± 1.1	0.994
BMI (kg/m^2^)	22.9 ± 0.4	34.5 ± 0.9	<0.001
Waist circumference (cm)	78.6 ± 2.2	105.1 ± 4.2	<0.001
Hip circumference (cm)	95.0 ± 2.1	125.2 ± 7.3	<0.001
Waist-to-hip ratio	0.83 ± 0.02	0.86 ± 0.05	0.538
Visceral fat mass (g)	350 ± 88	1,272 ± 140	<0.001
Abdominal fat mass (kg)	9.91 ± 0.98	23.22 ± 1.91	<0.001
Leg fat mass (kg)	7.57 ± 0.60	15.29 ± 1.18	<0.001
Fasting glucose (mmol/l)	4.97 ± 0.10	5.16 ± 0.20	0.404
2-Hour glucose (mmol/l)	5.09 ± 0.20	4.99 ± 0.30	0.775
Fasting insulin (pmol/l)	25.40 ± 4.00	48.60 ± 12.90	0.053
HOMA2-IR	0.47 ± 0.1	0.92 ± 0.3	0.050
SBP (mmHg)	120.9 ± 3.9	131.3 ± 4.3	0.098
DBP (mmHg)	76.2 ± 3.1	80.6 ± 3.1	0.356

BMI, body mass index; DBP, diastolic blood pressure; HOMA2-IR, Homeostasis Model Assessment 2–Insulin Resistance; SBP, systolic blood pressure. Data are mean ± SEM.

### 3.2 Plasma adipokine concentrations

**Figure 1:**
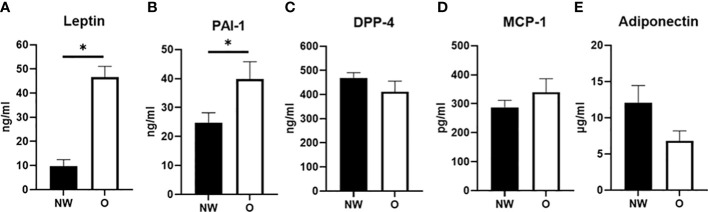
Plasma adipokine concentrations in arterial blood from postmenopausal women with normal weight (n = 9) and obesity (n = 6). **(A)** Leptin, **(B)** plasminogen activator inhibitor (PAI-1), **(C)** dipeptidyl-peptidase (DPP)-4, **(D)** monocyte chemoattractant protein (MCP-1), and **(E)** adiponectin; NW, normal weight; O, obesity. Data are expressed as mean ± SEM. *p < 0.05.

Arterial plasma concentrations of adipokines were measured after an overnight fast (**Figure 1**). Plasma leptin concentrations were significantly higher in women with obesity compared to normal weight (46.6 ± 3.1 *vs*. 9.8 ± 1.8 ng/ml, respectively, p < 0.001) (**Figure 1A**). Furthermore, PAI-1 concentrations were higher in individuals with obesity than normal weight (39.8 ± 4.3 *vs*. 24.8 ± 2.4 ng/ml, respectively, p = 0.036) (**Figure 1B**). No significant differences were found for circulating DPP-4 (412.0 ± 30.1 *vs*. 469.4 ± 15.4 ng/ml, p = 0.272) and MCP-1 concentrations (339.6 ± 31.6 *vs*. 287.4 ± 17.1 ng/ml, respectively, p = 0.299) between women with obesity and normal weight (**Figures 1C, D**). Finally, a tendency for lower circulating adiponectin concentration in women with obesity compared to normal weight was found (6.8 ± 0.9 *vs*. 12.1 ± 1.6 µg/ml, respectively, p = 0.088) (**Figure 1E**). IL-6 concentrations were below the detection limit for most individuals and are therefore not reported.

### 3.3 *In vivo* secretion of adipokines from abdominal and femoral subcutaneous adipose tissue

**Figure 2:**
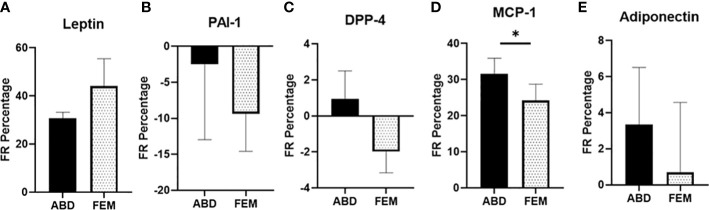
Fractional release of adipokines across subcutaneous abdominal (ABD) and femoral (FEM) subcutaneous adipose tissue (SAT) in postmenopausal women with normal weight (n = 9) and obesity (n = 6). **(A)** Leptin **(B)** PAI-1, **(C)** DPP-4, **(D)** MCP-1, and **(E)** adiponectin; paired data from ABD and FEM SAT are shown. Data are expressed as mean ± SEM. *p < 0.05.

To explore whether *in vivo* adipokine release is different across ABD and FEM SAT, we directly measured the fractional release (FR) of several adipokines in women with obesity or normal weight using the arterio-venous balance technique (**Figure 2**). Significant FR was only found for leptin and MCP-1 (both p = 0.001 *vs*. zero release). Leptin FR was similar between ABD and FEM depots (30.7 ± 2.6 *vs*. 44.1 ± 11.4%, respectively, p = 0.383) (**Figure 2A**). The FR of MCP-1 across ABD SAT was significantly higher than that across FEM SAT (31.6% ± 4.4% *vs*. 24.2% ± 4.5%, respectively, p = 0.023) (**Figure 2D**).

### 3.4 Abdominal and femoral subcutaneous adipose tissue blood flow

Pooled data from women with normal weight and obesity demonstrated that fasting ATBF was not significantly different between ABD and FEM SAT (9.3 ± 2.1 versus 5.8 ± 1.8 ml/min, p = 0.296). More specifically, there were also no significant differences between fasting ABD and FEM ATBF in women with normal weight (p = 0.641, *n =* 8) and obesity (p = 0.313, *n =* 6). Furthermore, ABD ATBF (8.4 ± 2.1 *vs*. 11.2 ± 2.9 ml/min, respectively, p = 0.459) and FEM ATBF (5.3 ± 1.5 *vs*. 6.4 ± 2.1 ml/min, respectively, p = 0.755) were not significantly different between women with normal weight and obesity.

### 3.5 Abdominal and femoral adipocyte morphology

**Figure 3:**
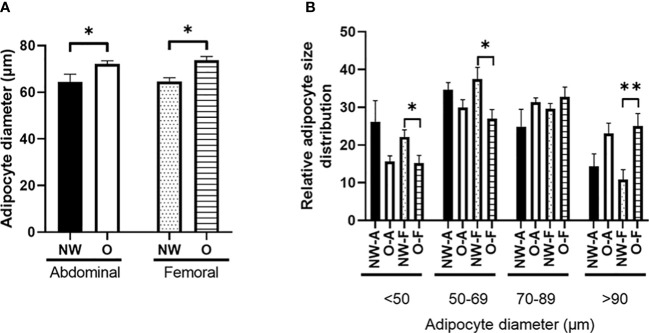
Morphology of subcutaneous adipocytes from individuals with normal weight (n = 11) and obesity (n = 8). **(A)** Fat cell size; **(B)** relative adipocyte size distribution (%). NW-A, normal weight ABD; O-A, obese abdominal; NW-F, normal weight FEM; O-F, obese FEM. NW, normal weight; O, obesity. Data are expressed as mean ± SEM. *p < 0.05, **p < 0.001.

Adipocytes from women with normal weight were significantly smaller compared to adipocytes from women with obesity, both for ABD (p = 0.014) and FEM SAT (p = 0.001) (**Figure 3A**). The smaller mean adipocyte size of ABD and FEM SAT in normal-weight individuals was explained by a lower frequency of very large adipocytes and a higher frequency of very small adipocytes as compared to women with obesity (**Figure 3B**). Pooled data from women with normal weight and obesity showed that adipocyte size was not different between ABD and FEM SAT (68.5 ± 1.9 versus 68.4 ± 1.6 μm, p = 0.791). In line with this, no significant differences in adipocyte size were found between ABD and FEM SAT in women with normal weight (p = 0.730) and obesity (p = 1.000).

### 3.6 Abdominal and femoral subcutaneous adipose tissue gene expression

**Figure 4:**
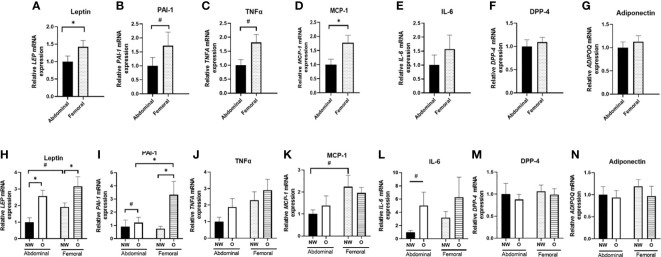
Gene expression of adipokines in ABD and FEM subcutaneous adipose tissue. Data are shown for the total group of women with normal weight and obesity (**A–G**; pairs n = 18) as well as for the normal weight and obese groups separately (**H–N**; ABD NW, n = 10, ABD O n = 9, FEM NW n = 11, FEM O n = 9). NW, normal weight; O, obesity. Data are expressed as mean ± SEM. *p < 0.05, # p < 0.01.

Next, we assessed the adipokine gene expression profile in ABD and FEM SAT (**Figures 4A–G**). Pooled data from women with normal weight or obesity showed that gene expression of leptin (p = 0.010) and MCP-1 (p = 0.027) was significantly lower in ABD than FEM SAT, while a tendency for lower PAI-1 (p = 0.080) and tumor necrosis factor-α (TNF-α; p = 0.090) gene expression in ABD compared to FEM was found. No significant differences in gene expression of IL-6, DPP-4, and adiponectin were found between ABD and FEM SAT.

Furthermore, we compared adipokine gene expression in women with normal weight and obesity separately (**Figures 4H–N**). ABD (p = 0.002) and FEM (p = 0.046) SAT gene expression of leptin were significantly higher in women with obesity than normal weight. Furthermore, ABD (p = 0.095) and FEM SAT (p = 0.014) gene expression of PAI-1 were higher in obesity. In addition, ABD SAT gene expression of IL-6 (p = 0.053) tended to be higher in women with obesity than normal weight. When examining SAT depot-differences in normal weight and obese groups separately, we found a significantly lower PAI-1 gene expression in ABD than FEM SAT in women with obesity (p = 0.008). Moreover, leptin (p = 0.052) and MCP-1 (p = 0.075) gene expression tended to be lower in ABD than FEM SAT in individuals with normal weight. No significant SAT depot-differences in adiponectin, DPP-4, and TNF-α gene expression were found in individuals with normal weight and obesity.

We found significant SAT depot-specific correlations between fat cell size and gene expression levels. Leptin gene expression was positively correlated with fat cell size both in ABD (r = 0.657; p = 0.024) and FEM SAT (r = 0.515; p = 0.024), while PAI-1 gene expression in FEM SAT was positively correlated with FEM fat cell size (r = 0.690; p = 0.001) but PAI-1 gene expression in ABD AT was not significantly associated with ABD fat cell size (r = 0.385, p = 0.218). No significant correlations between fat cell size and gene expression levels of IL-6, TNF-α, DPP-4, MCP-1, and adiponectin were found (data not shown).

### 3.7 Gene expression in differentiated human multipotent adipose-derived stem from abdominal and femoral subcutaneous adipose tissue

**Figure 5:**
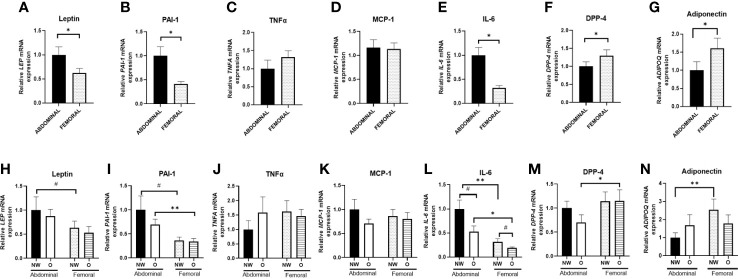
Adipokine gene expression in adipose tissue–derived mesenchymal stem cells that were differentiated for 14 days. Data are shown for the total group of women with normal weight and obesity (**A–G**; n = 18) as well as for both groups separately (**H–N**; ABD NW, n = 9; ABD O, n = 9; FEM NW, n = 9; FEM O, n = 9). NW, normal weight; O, obesity. Data are expressed as mean ± SEM. *p < 0.05, **p < 0.001, # p < 0.01.

Since AT consists of multiple cell types (3), including immune cells, we next specifically examined gene expression in differentiated hMADS derived from ABD and FEM SAT (**Figures 5A–G**) obtained from the same individuals with normal weight or obesity that underwent *in vivo* measurements and SAT biopsies (**Figure 5**). Pooled data from women with normal weight or obesity showed that gene expression of leptin (p = 0.009, **Figure 5A**), PAI-1 (p < 0.001, **Figure 5B**), and IL-6 (p < 0.001, **Figure 5E**) were significantly higher in ABD compared to FEM adipocytes. ABD adipocytes showed a lower gene expression of DPP-4 (p = 0.035, **Figure 5F**) and adiponectin (p = 0.029, **Figure 5G**). No significant differences between ABD and FEM adipocytes were observed for TNF*-*α (p = 0.284, **Figure 5C**) and MCP-1 (p = 0.712, **Figure 5D**) gene expression. Gene expression of the adipocyte differentiation markers PPARγ, C/EBPα, PLIN1, and FAS was not significantly different between ABD and FEM adipocytes (**Supplementary Materials—Figure 1**).

Furthermore, we compared adipokine gene expression in differentiated adipocytes from women with normal weight or obesity separately (**Figures 5H–N**). We found a higher gene expression of IL-6 and PAI-1 in ABD compared to FEM adipocytes derived from individuals with normal weight (p = 0.006 and p = 0.068, respectively) and obesity (p = 0.018 and p = 0.002, respectively) (**Figures 5L, I**). Furthermore, ABD adipocytes derived from normal-weight women showed lower adiponectin (p = 0.005, **Figure 5N**) and higher leptin (p = 0.098) gene expression compared to FEM adipocytes (**Figure 5H**). In addition, DPP-4 gene expression (**Figure 5M**) was significantly lower in ABD than FEM adipocytes derived from women with obesity (p = 0.043). No adipocyte depot-differences were found for TNF*-*α and MCP-1 gene expression (**Figures 5J, K**). Finally, IL-6 gene expression tended to be higher in both ABD (p = 0.054) and FEM (p = 0.069) adipocytes derived from women with normal weight compared to obesity (**Figure 5L**).

### 3.8 Adipokine secretion from differentiated abdominal and femoral human multipotent adipose-derived stem

**Figure 6:**
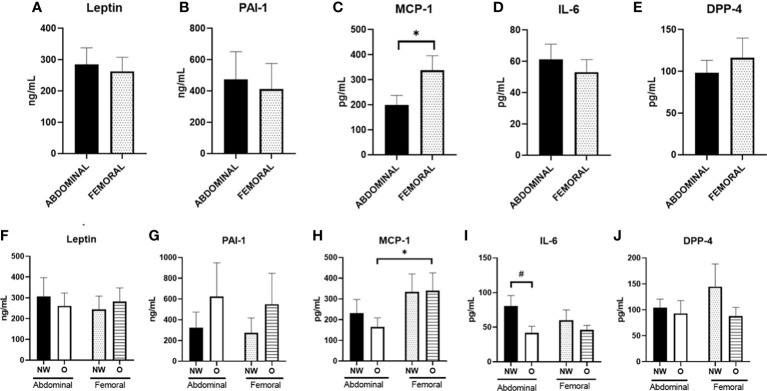
Adipokine secretion from adipose tissue–derived mesenchymal stem cells that were differentiated for 14 days. Data are shown for the total group of women with normal weight and obesity (**A–E**; n = 18) as well as for both groups separately (**F–J**); ABD NW, n = 9; abdominal O, n = 9; FEM NW, n = 9; FEM O, n = 9). NW, normal weight; O, obesity. Data are expressed as mean ± SEM. *p < 0.05, # p < 0.01.

Finally, we investigated the secretion of adipokines from human primary ABD and FEM adipocytes (**Figure 6**). Pooled data from women with normal weight or obesity showed significantly lower secretion of MCP-1 from ABD compared to FEM adipocytes (198.5 ± 39.1 pg/ml versus 337.6 ± 58.5 pg/ml, p = 0.004) (**Figure 6C**). No significant depot-differences in secretion rates of leptin, PAI-1, IL-6, and DPP-4 between ABD and FEM adipocytes were present.

When comparing adipokine secretion from ABD and FEM adipocytes from women with normal weight or obesity separately, we found a significantly lower secretion of MCP-1 from ABD compared to FEM adipocytes derived from women with obesity (165 ± 44 *vs*. 340 ± 85 pg/ml, p = 0.013) (**Figure 6H**). No significant depot-differences in secretion rates of leptin, PAI-1, IL-6, DPP-4, and MCP-1 between ABD and FEM adipocytes were present. In addition, the secretion of IL-6 from ABD adipocytes tended to be higher in cells derived from women with normal weight compared to obesity (80.5 ± 15.2 pg/ml versus 41.8 ± 9.4 pg/ml, respectively, p = 0.063) (**Figure 6D**). Adiponectin secretion was below the detection limit, and these data are therefore not reported.

## 4 Discussion

In the present study, we investigated the inflammatory signatures of ABD and FEM SAT in postmenopausal women with normal weight and obesity. More specifically, we compared the *in vivo* release of adipokine from ABD and FEM SAT in both groups, examined adipocyte morphology and gene expression of adipokines in these SAT depots, and determined gene expression and secretion of adipokines *in vitro* using differentiated human primary ABD and FEM subcutaneous adipocytes derived from the same study participants. The present findings demonstrate for the first time that upper and lower body adipose tissue as well as adipocytes are characterized by distinct inflammatory signatures in postmenopausal women with normal weight and obesity.

In the present study, we assessed leptin and adiponectin as classical adipokines altered in obesity (36–39) and determined the expression and secretion of several well-known proinflammatory molecules (TNF-α, IL-6, PAI-1, DPP-4, and MCP-1) that have been linked to obesity and cardiometabolic disease risk (2, 3, 40–49). We found significant fractional release of leptin and MCP-1 from ABD and FEM subcutaneous SAT, with similar fractional release of leptin from both SAT depots and higher release of MCP-1 from ABD compared to FEM SAT. The comparable release of leptin from FEM and ABD SAT is in line with a previous report (23). No release of other adipokines, including PAI-1, DPP-4, and adiponectin, across ABD and FEM SAT was detectable. The latter is in line with previous studies, showing no significant release of adiponectin, IL-6, and DPP-4 across human ABD SAT in people with normal weight and obesity (48, 50). One study that did report *in vivo* DPP-4 release across human ABD SAT only found significant release in few individuals with low (<288 ng/ml) plasma DPP-4 concentrations (48), while mean DPP-4 concentrations were much higher (>400 ng/ml) in the present study. The lack of detectable adiponectin release across SAT may be explained by a low release rate and long half-life, reflected by relatively constant circulating concentrations of these adipokines (8). We found higher arterial concentrations of leptin and PAI-1 in women with obesity. Since no differences in the *in vivo* fractional release of these factors from ABD and FEM SAT were found between individuals with normal weight and obesity, the higher-circulating leptin and PAI-1 concentrations are likely explained by the higher total fat mass in obesity.

Differences in the functional properties between AT depots may underlie the cardiometabolic disease risk associated with a certain body fat distribution pattern. Indeed, functional differences between ABD and FEM SAT seem to emerge from adipocytes having distinct properties (4, 9). Many studies have demonstrated a close relationship between adipocyte morphology and AT function, with hypertrophic adipocytes (as often seen in people with obesity) showing impairments in lipid metabolism and a more proinflammatory phenotype, which may aggravate insulin resistance (1, 3, 4, 51). In the present study, women with obesity had larger adipocytes than individuals with normal weight, both in ABD and FEM SAT. However, adipocyte size did not differ between ABD and FEM SAT in both groups. This is in agreement with some (23, 52, 53) but not all previous reports comparing upper and lower body SAT (33, 54–56), and may relate to characteristics of the study populations investigated (i.e., age and metabolic status). Our study participants did not have severe obesity and had, by definition for inclusion in the study, a relatively healthy metabolic profile. In line with adipocyte hypertrophy in women with obesity, we found higher SAT gene expression of leptin, PAI-1, and IL-6 (only in ABD SAT) in the people with obesity. Few studies, however, have directly compared upper and lower body SAT inflammation. Intriguingly, despite similar fat cell sizes in both SAT depots, the present findings demonstrate lower gene expression of leptin, MCP-1, PAI-1, and TNF-α in ABD than FEM SAT. Previous reports indicated that lower body SAT shows a similar (24) or more proinflammatory profile compared to ABD SAT (57). Moreover, global transcriptional profiling of men and women failed to identify differentially expressed clusters of inflammation-specific genes between ABD and gluteal SAT, although stronger associations between the expression of proinflammatory factors and several obesity-related traits were found for ABD SAT (23).

Since whole-AT gene expression profiles are determined by gene expression in multiple adipose-derived cell types such as adipocytes and immune cells, we also specifically investigated gene expression profiles in differentiated human primary ABD and FEM subcutaneous adipocytes derived from the participants that underwent the *in vivo* measurements and SAT biopsies. Interestingly, we observed that hMADS cells derived from ABD and FEM SAT that have been differentiated *in vitro* (and therefore been exposed to the same experimental microenvironment) show different gene expression patterns. Indeed, we demonstrate higher gene expression of the proinflammatory factors IL-6 and PAI-1 in ABD compared to FEM adipocytes derived from women with both normal weight and obesity. Furthermore, the expression of leptin was higher and that of adiponectin lower in ABD compared to FEM adipocytes derived from women with normal weight. These findings highlight intrinsic differences in the inflammatory signatures of human ABD and FEM adipocytes, which are already present in cells derived from a healthy (‘non-obese’) AT microenvironment (i.e., normal-weight individuals). In addition, DPP-4 gene expression was lower in ABD than FEM adipocytes derived from women with obesity. Adipocyte differentiation markers were not significantly different between ABD and FEM adipocytes, suggesting that these differences in adipocyte gene expression are not due to differences in adipocyte differentiation between ABD and FEM adipocytes. The fact that inflammatory gene expression was not higher in differentiated human primary ABD and FEM adipocytes derived from women with obesity compared to normal weight provides further support for the notion that adipocyte hypertrophy and/or the contribution of the inflammatory cell component are key factors determining the *in vivo* AT inflammatory signature. The differences in adipocyte gene expression did, however, not translate into functional differences in the secretion of adipokines. Specifically, we only found a lower secretion of MCP-1 from ABD compared to FEM adipocytes derived from both women with normal weight or obesity, but no differences in the secretion rates of leptin, PAI-1, IL-6, and DPP-4 between ABD and FEM adipocytes were apparent. The discrepancy between MCP-1 fractional release *in vivo* being higher from ABD versus FEM SAT, while ABD SAT MCP-1 gene expression as well as ABD adipocyte MCP-1 secretion were lower compared to FEM SAT/adipocytes might be explained by depot-differences in post-transcriptional regulation and secretory pathways influencing the release of adipokines from these fat depots. Notably, gene expression of IL-6 was higher in ABD than FEM adipocytes, while no significant differences in IL-6 gene expression were found between ABD and FEM AT. This might be explained by depot-differences in IL-6 expression due to the presence of other cells than adipocytes such as immune cells (58), which warrants further investigation.

A strength of the present study is that we, for the first time, combined paired *in vivo* measurements across ABD and FEM SAT, analyses in ABD and FEM SAT biopsies, and *in vitro* experiments using differentiated human primary ABD and FEM subcutaneous adipocytes derived from the study participants. Furthermore, we did not perform the experiments using a pool of stem cells from normal-weight and obese donors (risking those outcomes are influenced/masked by strong effects seen in a specific donor) or a single donor, as often done, but performed the *in vitro* experiments with cells from many donors with normal weight and obesity separately.

Noteworthy, the present study also has some limitations. First, a formal power calculation was not performed, given the exploratory nature of the study. The number of participants we aimed to include in our study to detect differences was based on previous studies using the arterio-venous balance technique to investigate group differences in adipokines/metabolites across upper-body versus lower-body AT (25). Third, due to the technical difficulties to cannulate and collect blood samples from the small veins in ABD and FEM, we were able to successfully complete sample collection for nine women with normal weight and six women with obesity. Due to the limited number of paired blood samples draining ABD and FEM SAT for the individuals with normal weight and obesity, we could unfortunately not analyze data separately for both groups due to limited statistical power. Secondly, we determined ATBF using Doppler ultrasound, which provides data on intravascular blood flow in relatively large SAT veins rather than at the capillary level (32). Unfortunately, it was not possible to utilize the ^133^Xe wash-out technique due to the global production stop of medical ^133^Xe (32). Consequently, we could not quantify absolute fluxes of adipokines per unit AT, and data on *in vivo* release of adipokines should therefore be interpreted with some caution. Nevertheless, calculation of fractional release of adipokines also yields valuable insights into adipokine release across different AT depots, especially since ATBF was not significantly different between ABD and FEM SAT in the present study. Finally, we only studied the superficial layer of SAT. Previous studies have shown different functional properties when comparing adipocytes derived from the superficial and deep subcutaneous layers (59).

In conclusion, our findings demonstrate that upper and lower body SAT are characterized by distinct inflammatory signatures in postmenopausal women with normal weight and obesity, which seem independent of adipocyte size. Future studies with a larger sample size are warranted to investigate functional differences of upper and lower body SAT in different populations, taking age, sex, metabolic status, body composition, obesity duration and weight cycling, as well as differential immune cell populations into account, and relate these to metabolic health at the whole-body level.

## Data availability statement

The original data of the present study are included in the article/**Supplementary Material**. Further inquiries related to raw data can be directed to the corresponding author.

## Ethics statement

The studies involving human participants were reviewed and approved by University of Birmingham Ethics committee and the UK Health Research Authority National Health System Research Ethics Committee. The patients/participants provided their written informed consent to participate in this study.

## Author contributions

KM and GG acquired funding, conceived, and designed research, interpreted data, and revised the manuscript. IL performed experiments, analyzed data, interpreted data, prepared figures, and drafted the manuscript. NH, YE, JJ, and RD performed experiments and analyzed data. EB and JJ interpreted data and revised the manuscript. All authors contributed to the article and approved the submitted version.
