# Supplementary material for: Distinct inflammatory signatures of upper and lower body adipose tissue and adipocytes in women with normal weight or obesity
Source: Front Endocrinol (Lausanne). 2023 Jun 26;14:1205799. doi: 10.3389/fendo.2023.1205799 (PMC10338223; doi:10.3389/fendo.2023.1205799)
Supplement: Supplementary file 1 [file DataSheet_1.docx]

Supplementary Material

Distinct inflammatory signatures of upper and lower body adipose tissue and adipocytes in women with normal weight or obesity

Ioannis G. Lempesis, Nicole Hoebers, Yvonne Essers, Johan W.E. Jocken, Rosemary Dineen, Ellen E. Blaak, Konstantinos N. Manolopoulos, Gijs H. Goossens*

*** Correspondence:** Dr. Gijs H. Goossens, PhD. G.Goossens@maastrichtuniversity.nl

# Supplemental methods

## Human primary adipocyte experiments

We determined gene expression of several adipocyte differentiation markers: *peroxisome proliferator-activated receptor γ (PPARγ), CCAAT-enhancer binding protein α (C/EBPα), fatty acid synthase (FAS), and Perilipin 1 (PLIN1)*. Total RNA was extracted from hMADS cells using TRIzol reagent (Invitrogen, Breda, Netherlands), and SYBR-Green–based real-time PCRs were performed using an iCycler (Bio-Rad, Veenendaal, Netherlands). Results were normalized to the mean of 18S ribosomal RNA.

# Supplemental Table 1:

Primer sequences

|  |  | **Sequence** |
| --- | --- | --- |
| TNF-α | Forward | CCGAGTGACAAGCCTGTAGC |
|  | Reverse | GAGGACCTGGGAGTAGATGAG |
| IL-6 | Forward | AAATTCGGTACATCCTCGACGG |
|  | Reverse | GGAAGGTTCAGGTTGTTTTCTGC |
| DPP-4 | Forward | AGTGGCGTGTTCAAGTGTGG |
|  | Reverse | CAAGGTTGTCTTCTGGAGTTGG |
| PAI-1 | Forward | TCGTCCAGCGGGATCTGAA |
|  | Reverse | GCCGTTGAAGTAGAGGGCATT |
| MCP-1 | Forward | CCCCAGTCACCTGCTGTTAT |
|  | Reverse | TCCTGAACCCACTTCTGCTT |
| Adiponectin | Forward | TGGTGAGAAGGGTGAGAA |
|  | Reverse | GTTCAGTCCACAGTGTCGCAGA |
| Leptin | Forward | GCTGTGCCCATCCAAAAAGTCC |
|  | Reverse | CCCAGGAATGAAGTCCAAACCG |
| 18S | Forward | AGTTAGCATGCCAGAGTCTCG |
|  | Reverse | TGCATGGCCGTTCTTAGTTG |
| PPARγ | Forward | TACTGTCGGTTTCAGAAATGCC |
|  | Reverse | GTCAGCGGACTCTGGATTCAG |
| FAS | Forward | CCGAGACACTCGTGGGCTA |
|  | Reverse | CTTCAGCAGGACATTGATGCC |
| PLIN1 | Forward | CTCTCGATACACCGTGCAGA |
|  | Reverse | TGGTCCTCATGATCCTCCTC |
| VEGFA | Forward | TCCGGGCTCGGTGATTTA |
|  | Reverse | GACTCCGGCGGAAGCAT |
| GLUT1 | Forward | GATTGGCTCCTTCTCTGTGG |
|  | Reverse | TCAAAGGACTTGCCCAGTTT |
| BNIP3 | Forward | ATCAAAAGGTGCTGGTGGAG |
|  | Reverse | ACCCTCAGCATGAGGAACAC |
| C/EBPα | Forward | AAGAAGTCGGTGGACAAGAACAG |
|  | Reverse | GCGGTCATTGTCACTGGTCA |

# Supplementary Figures


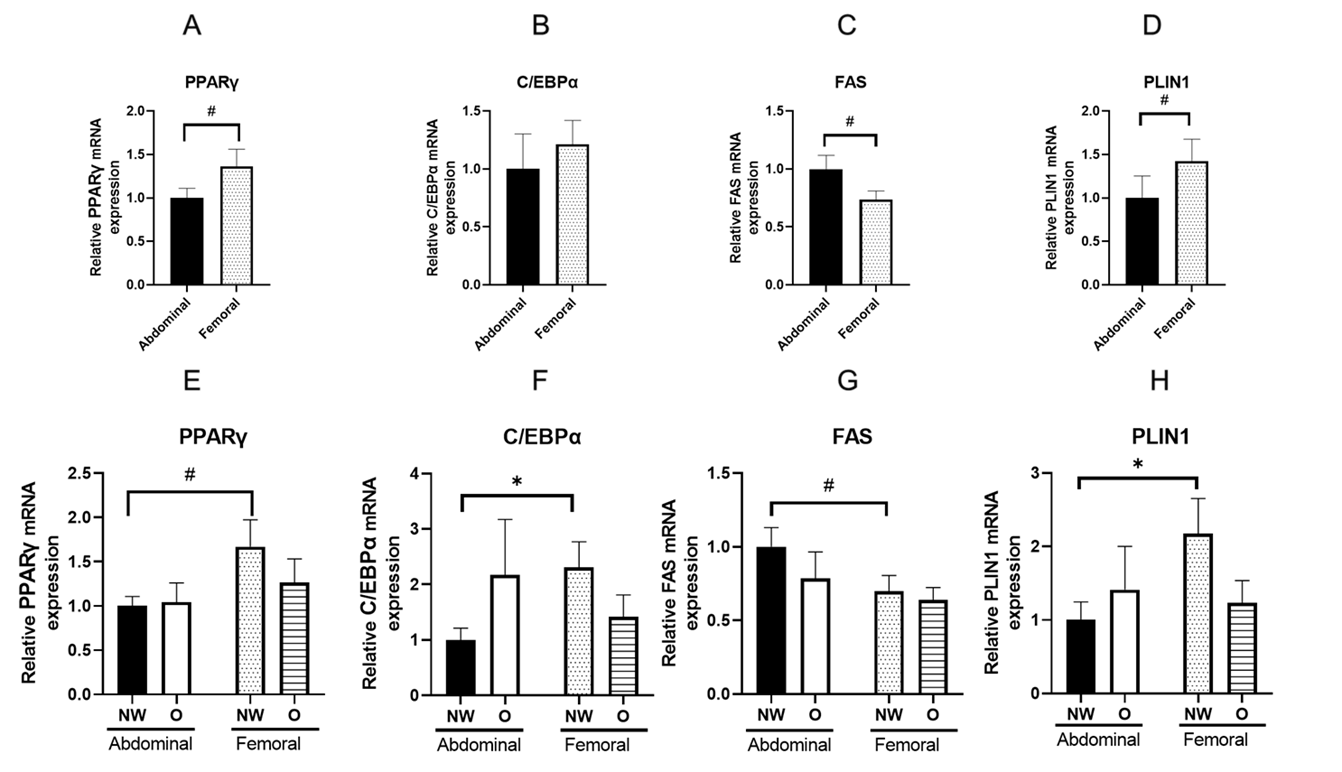


**Supplementary Figure 1.** Gene expression of adipocyte differentiation markers in adipose tissue-derived mesenchymal stem cells that were differentiated for 14 days. Data are shown for the total group of women with normal weight and obesity (panels A – D; n = 18) as well as for both groups separately (Panels E – H; abdominal NW, n = 9; abdominal O, n = 8; femoral NW, n = 9; femoral O, n = 9). *C/EBPα, CCAAT-enhancer binding protein α; FAS fatty acid synthase*; NW, normal weight; O, obesity; *PLIN1, Perilipin 1; PPARγ, peroxisome proliferator-activated receptor γ*. Data are expressed as mean ± SEM. *p<0.05, #p<0.10.


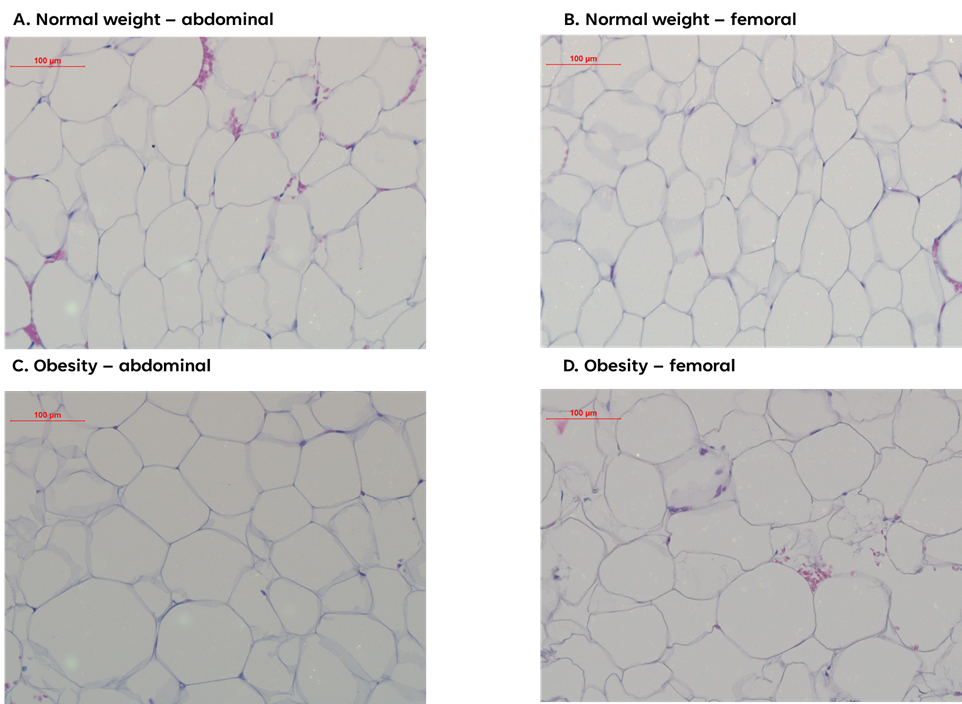


Supplementary Figure 2. Representative images with Haemotoxylin and Eosin (H&E) staining from adipose tissue biopsies from individuals with normal weight (panels A and B) or obesity (panels C and D) from abdominal subcutaneous adipose tissue (panels A and C) and femoral subcutaneous adipose tissue (B and D). Images indicate the differences between BMI groups with simple visual inspection. Nuclei are stained purple/blue while collagen, cell cytoplasm, erythrocytes shades of pink red. The adipocyte area is represented by the empty-like white space, where the lipids accumulate.
